# The prevalence of disability in older adults with multimorbidity: a meta-analysis

**DOI:** 10.1007/s40520-024-02835-2

**Published:** 2024-09-10

**Authors:** Jin Zhang, Yan Sun, Aiying Li

**Affiliations:** 1 Sichuan Academy of Medical Sciences & Sichuan Provincial People’s Hospital, Chengdu, Sichuan China; 2 School of Nursing, Chengdu, Chengdu University of Traditional Chinese Medicine, Chengdu, Sichuan China

**Keywords:** Older adults, Multimorbidity, Disability

## Abstract

**Background:**

Disability is typically correlated with lower quality of life and decreased capacity for self-care. It has been demonstrated that multimorbidity is closely linked to a variety of unfavorable events, such as disability. Researchers are still figuring out how and to what extent co-morbidities impact disability, though. In order to fill up this gap, this study examines the prevalence and contributing variables of disability in older patients who have multimorbidity.

**Methods:**

We conducted a systematic search of Pubmed, Cochrane Library, Web of Science, Embase, and CINAL databases for articles from their inception until September 2023. We selected co-morbid older adults aged > 60 years and used the ADL scale or any scale that assesses disability as an assessment tool. We excluded literature that did not meet the criteria, and literature that could not be included in the data we needed. We extracted data from the included literature and calculated synthetic prevalence rates, ORs, and 95% confidence intervals.

**Results:**

A total of 32 papers (71,135 older adults) were included in the study. The prevalence of disability among older patients with multimorbidity was around 34.9% (95% CI = 25.8-43.9%). Subgroup analysis showed higher rates of disability among comorbidities who were older, female, unmarried, and long-term users of health services. And the incidence of disability increased each year. Meanwhile, the regions of the United States, China, and Spain showed higher rates of disability.

**Conclusions:**

Disability rates in older patients with multimorbidity are higher, thus it’s critical to focus on risk factors while fully accounting for regional variances.

**Supplementary Information:**

The online version contains supplementary material available at 10.1007/s40520-024-02835-2.

## Introduction

Multimorbidity, also known as multiple chronic diseases(MCC), refers to the simultaneous presence of two or more chronic diseases in an individual [1, 2]. Due to improvements in global healthcare, a rise in the average lifespan, and demographic shifts, multimorbidity is becoming more common. The prevalence of multimorbidity is approximately 30% in the young and middle-aged population [3], it can reach up to 55−93% in the population aged 60 and over [4]. Moreover, the prevalence and number of chronic diseases also appear to increase significantly with age, and multimorbidity is more common than any single chronic disease, with prevalence rates of more than 80% of the elderly population aged 80 and over [5].

Multimorbidity makes maintaining the health of older persons more challenging, especially the disabilities that accompany multimorbidity. Disability is a complicated multifactorial state that arises from the interplay of the individual with their whole environment. Disability means a reduction in the person’s autonomy and independence as well as their general capacity to adapt to their social and living environment. According to the World Health Organization’s (WHO) International Classification of Functioning, Disability, and Health (ICF) criteria, disability is often considered an impairment, activity restrictions, and participation limitation in general [6].

Multimorbidity and disability are strongly related, as has been shown in earlier research [7]. Physical functionality may be a good indicator of the cumulative combined impact of chronic illnesses on health. For each additional disease, the patient’s risk of dysfunction increases by 16%, life expectancy decreases by 1.8 years, and the risk of needing the assistance of a healthcare professional increases by 20% [8, 9]. These relationships are typically nonlinear, multimorbidity accelerating the weakening of bodily functions and leading to a rapid deterioration of an individual’s metabolic mechanisms, and given the physiological vulnerabilities associated with aging, older adults are more likely to experience functional disability. The healthcare system is put under an additional burden because of the relationship between disabilities and adverse outcomes including falls and depression, a considerable increase in mortality, frailty, and Emergency Department visits, and a higher likelihood that patients may face social and economic squalor in later life [10–13]. In order to effectively address the disability of patients, minimize eventual healthcare expenses, and enhance health, determining the disability rate of patients with multimorbidity as well as the risk factors has thus become especially crucial.

Currently, only one systematic review [14] from 2015 explored the correlation between multimorbidity and functional decline. However, the study had a large age span (> 18 years) and did not focus on older adults who were most affected by comorbidity; On the other hand, the study did not calculate the combined rate of disability, and explored and analyzed the influencing factors. Moreover, the study has a long history, and it needs to be discussed in newer literature.Therefore, this study explores and explains this part of the content, and provides a basis for future intervention to delay and control the progress of disability.

## Methods

### Materials and methods

This study has been registered with the International Prospective Register of Systematic Reviews (Prospero) under the registration number CRD42023425740. This article is reported according to Preferred Reporting Items for Systematic Reviews and Meta-Analysis (PRISMA).This study was based on the Cochrane Handbook of Systematic Literature Reviews.

### Search strategy

Two researchers searched the literature from the PubMed, Cochrane Library, EMBASE, CINAL, and Web of Science databases with an English-only search language from the library until September 2023. We used Medical Subject Headings (MeSH) keywords to search by title and abstract, using AND, OR to concatenate the following text: older adults (older population, aged, elderly, geriatric) AND multimorbidity (multiple chronic conditions) AND disability (activities of daily living, disabled, ADL, BADL, IADL ). Additional articles were then incorporated by way of hand-searched references.

### Eligibility criteria

Inclusion criteria: (1) age 60 and above; (2) multimorbidity: refers to the simultaneous presence of two or more chronic diseases in an individual; (3) studies reporting clinical outcomes including disability. Activities of Daily Living Scale (ADL) are the most common indicator of disability and includes the Basic Activities of Daily Living Scale (BADL) and Instrumental Activities of Daily Living Scale (IADL). The most common scales for BADL include the Katz Independence Index and the Barthel Index, with 6 or 10 items. Including dressing, eating, bathing, dressing, etc. while the IADL has 8 items, including shopping, cooking, etc. In addition, any literature that could define an indicator of disability was also included. (4) study design: Observational studies, including cross-sectional and cohort studies; (5) using disability as a dichotomous outcome indicator, data from the literature provided corrected ORs and 95% CI or sufficient data to calculate prevalence.

The following papers are excluded: duplicates, unrelated to the topic, documents lacking the whole text, and documents from which the necessary data cannot be retrieved.

### Risk of bias assessment

Two investigators evaluated the quality of the included literature. The Agency for Healthcare Research and Quality (AHRQ) scale was utilized in the cross-sectional study [15], which has 11 entries with a yes or no answer, yes scoring 1 and no scoring 0, respectively. The final score was 0–3 for low quality literature, 4–7 for moderate quality literature, and 8–11 for high quality literature. The Newcastle Ottawa Scale (NOS) were utilized in the cohort study [16], which has 8 entries, with 0–3 being low quality literature, 4–6 being moderate quality literature, and ≥ 7 being high quality literature, with higher scores indicating better quality literature.

### Data extraction

Two researchers were responsible for including, screening and extracting literature according to the criteria. When there was disagreement, we consulted a third researcher. The extracted data was as follows: (a) basic characteristics: authors, year, country, age, gender, number of events, total population, and incidence rate; and (b) study design, research instrument, sample source, sample size, and marital status.

### Quality of evidence

In this study, the quality of evidence was evaluated using GRADE (Grading of Recommendations, Assessments, Development, and Evaluation), which consists of 5 evaluations: (a) risk of bias; (b) inconsistency; (c) indirectness; (d) imprecision; and (e) publication bias. The presence of one item was downgraded to a medium quality level; the presence of two items was downgraded to a low quality level; and the presence of three items was a very low quality level.

### Statistical analysis

The two researchers independently extracted the data into EXCEL and then analyzed the prevalence rate and 95% confidence interval by STATA17.0 software. The magnitude of heterogeneity was assessed using I ^2^ and Q tests [17, 18]. When *P* < 0.05 or I^2^ > 50%, it was considered that there was significant heterogeneity in the study, which was analyzed by the random-effects model (SMD); When *P* ≥ 0.5 or I^2^ ≤ 50%, it was considered that there was no significant heterogeneity, which was analyzed by the fixed-effects model (MD)The articles differed in nadir criteria, study design, and disability criteria, so there was significant heterogeneity between studies (I^2^ = 99.94%). Therefore, a random effects model was used to calculate the combined effect size. When studies had high heterogeneity, the source of heterogeneity was analyzed subgroups. The publication year (-2015, 2015–2020, 2020-), sample size (<500, > 500), study design (cross-sectional study, cohort study), data source (community, hospitals), gender (male > 50%, female > 50%), marital status (married, unmarried), and country were all taken into consideration. A sensitivity analysis was performed to assess the stability of the findings by excluding individual papers. Funnel plots were generated by Begg-Mazumdar’s and Egger’s tests to determine publication bias. Statistical significance was defined as *P* < 0.05, and 95% confidence intervals were calculated.

## Results

### Study selection

**Fig. 1:**
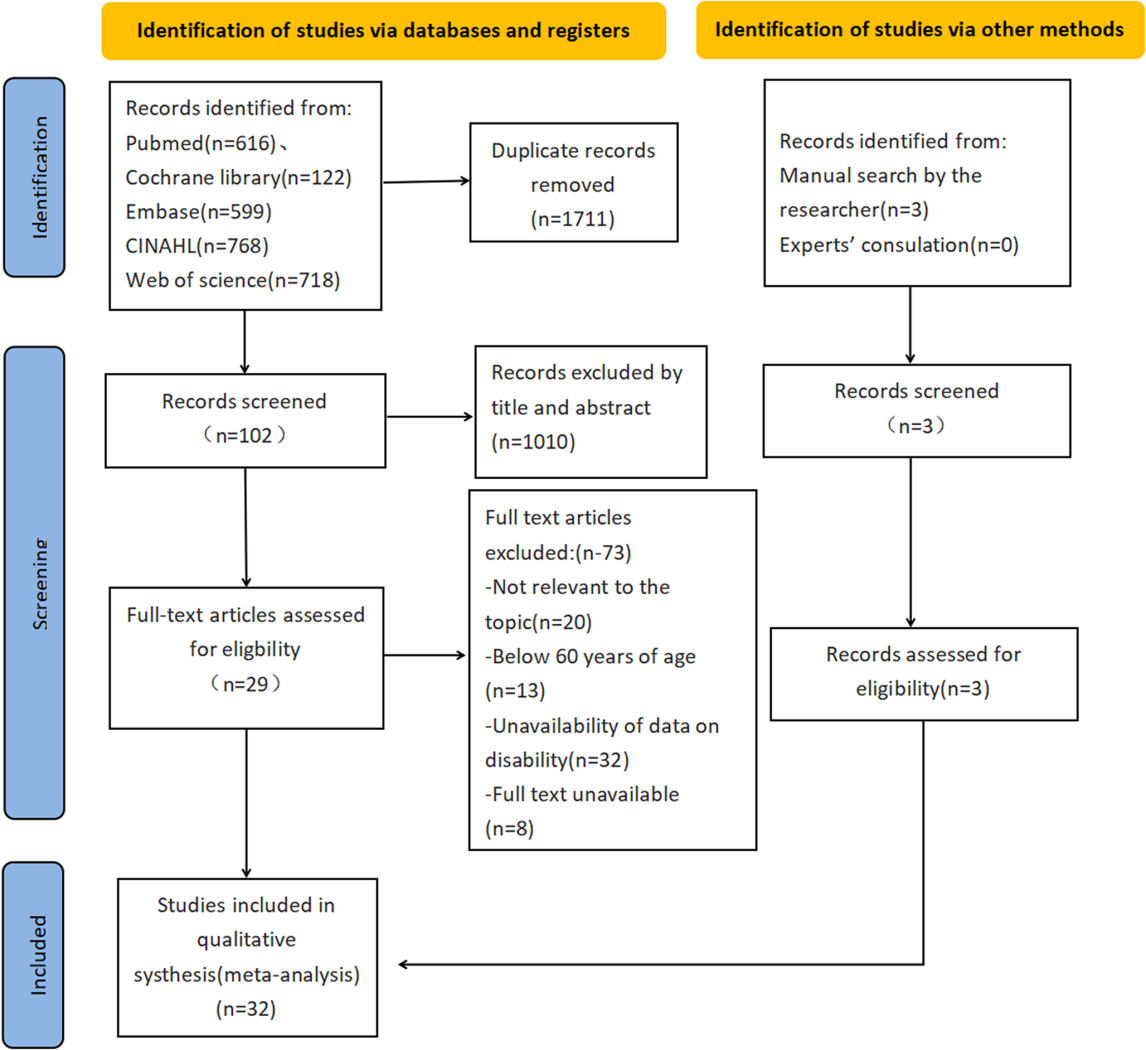
Flow diagram of studies selection

In this study, 2823 documents were retrieved from Pubmed, Cochrane Library, Web of Science, EMBASE, and CINAL, and 3 documents were searched manually, totaling 2826 documents included, and 1112 documents remained after deduplication. 1010 literatures were eliminated by reading titles and abstracts. Reading the remaining 102 literature, 73 of them were excluded because they did not meet the inclusion criteria. See Fig. 1.

### Study characteristics

32 studies with a total of 71,135 individuals were included in this study, with years from 2006 to 2023, covering countries such as Brazil (*n* = 1), China (*n* = 7), the United States (*n* = 5), Poland (*n* = 1), Sweden (*n* = 2), Spain (*n* = 4), Norway (*n* = 2), Belgium (*n* = 1), India (*n* = 1), Singapore (*n* = 1), South Korea (*n* = 1), Netherlands (*n* = 1), Canada (*n* = 1), Europe (*n* = 1), Iceland (*n* = 1), Nigeria (*n* = 1), Global (*n* = 1). See Table 1.

**Table 1:** Basic characteristics of the included literature

First author (year)	country	Study design	age	Total (male/female)	Study size	Disability (%)	Definition of disability	Sample source	Quality score	
Pivetta [19], 2020	Brazil	observational, cross-sectional study	>80	62	39	62.9%	ADL	community	8a	
Woo [20], 2014	China	Baseline of cohort study	≥ 65	2736	622	22.7%	IADL	community	6b	
Chamberlain [21], 2019	US	Cross-sectional study	>60	8120 (3604/4516)	2323	28.6%	ADL	Rochester Epidemiol- ogy Project (REP) medical records -linkage system.	9a	
Liu [22], 2021	China	Cross-sectional study	>60	352	311	88.4%	ADL	electronic health records of eight long-term care facilities.	8a	
Shirazi [23], 2023	US	Cross-sectional study	≥ 65	1508	788	52.3%	ADL	National Health and Aging Trends Survey (NHATS) baseline data	10a	
Shirazi [23], 2023	US	Cross-sectional study	≥ 65	3354	1735	51.7%	ADL	National Health and Aging Trends Survey (NHATS) baseline data	10a	
Bagai [24], 2022	global	Baseline of Cohort study	≥ 75	1872	948	50.6%	EQ−5D	stable coronary artery disease in post–myocardial infarction patients (TIGRIS) registry	7b	
Jędrzejczyk [25], 2022	Poland	Cross-sectional study	≥ 65	100 (48/52)	29	29.0%	ADL	inpatients	7a	
Marengoni [26], 2009	Sweden	Baseline of Prospective cohort study	>77	575	83	14.4%	ADL	community and institution	6b	
Abizanda [27], 2014	Spain	Baseline of Cohort study	≥ 70	512	142	27.7%	BADL	the FRADEA (Frailty and Dependence in Albacete) Study	8b	
Kshatri [28], 2020	India	Cross-sectional study	>60	354 (179/175)	43	12.1%	ADL	the rural block of Tigiria	7a	
Kshatri [28], 2020	India	Cross-sectional study	>60	(354) (179/175)	98	27.7%	IADL	the rural block of Tigiria	7a	
Marengoni [29], 2021	Sweden	Baseline of the Cohort study	>60	1558	355	22.8%	ADL	community or in institutions in the Kungshol-men central area of Stockholm	7b	
Marengoni [29], 2021	Sweden	Cohort study	>60	1558	276	17.7%	IADL	community or in institutions in the Kungshol-men central area of Stockholm	7b	
Schmidt [30], 2016	Europe	Cross-sectional study	>60	13,794 (5665/8129)	11,345	82.2%	ADL	the Survey of Health, Ageing and Retirement in Europe	6a	
Peng [31], 2020	China	Cross-sectional study	>60	589 (183/406)	96	16.3%	ADL	Random sampling from Foshan City and Shenzhen City in Guangdong Province	9a	
Peng [31], 2020	China	Cross-sectional study	>60	589 (183/406)	322	54.7%	IADL	Random sampling from Foshan City and Shenzhen City in Guangdong Province	9a	
Forjaz [32], 2015	Spain	Cohort study of Cross-sectional design	>65	705 (279/524)	181	25.6%	Barthel index/BADL	Quality of life in older adults–Spain study (CadeViMa )	7b	
Forjaz [32], 2015	Spain	Cohort study of Cross-sectional design	>65	443 (178/265)	315	71.1%	Specific disability measure (24 items)	Ageing in Spain Longitudinal Study, Pilot Survey(ELES )	7b	
Forjaz [32], 2015	Spain	Cohort study of Cross-sectional design	>65	4995 (1756/3239)	2571	51.5%	Specific disability measure (24 items)	National Health Survey in Spain(ENSE)	7b	
Lee [33], 2018	China	Cross-sectional study	≥ 65	4234 (1998/2236)	109	2.6%	Answer the question“Do you live independently”	Elderly Health Examination Database registrants	7a	
Lee [34], 2021	Korea	Baseline of cohort study	>65	554 (250/304)	25	4.5%	ADL	Six Waves of the Korean Longitudinal Study	7b	
Lee [34], 2021	Korea	Baseline of Cohort study	>65	554 (250/304)	105	19.0%	IADL	Six waves of the Korean Longitudinal Study	7b	
Laan [35], 2013	Netherland	Cross-sectional study	≥ 60	1187 (440/747)	706	59.5%	BADL/Katz−15	Three primary care networks	8a	
Wang [36], 2021	China	Cross-sectional study	≥ 60	1390	710	51.1%	BADL	long-term care insurance in Shanghai	9a	
Wang [36], 2021	China	Cross-sectional study	≥ 60	1390	1281	92.2%	IADL	long-term care insurance in Shanghai	9a	
Quan [37], 2017	Singapore	Cross-sectional study	≥ 65	498 (254/244)	132	26.5%	ADL	public primary care clinic	9a	
Quah [37], 2017	Singapore	Cross-sectional study	≥ 65	498 (254/244)	126	25.3%	IADL	public primary care clinic	9a	
Zhang [38], 2020	China	Cross-sectional study	≥ 60	3011 (1944/ 1068)	673	22.3%	ADL	12 tertiary hospitals from 7 provinces hospitalized patients	8a	
Martín [39], 2018	Spain	Baseline of Cohort study	≥ 65	241 (113/128)	31	12.9%	BADL	three primary care centers	7b	
Martín [39], 2018	Spain	Baseline of Cohort study	≥ 65	241 (113/128)	92	38.2%	IADL	three primary care centers	7b	
Martín [40], 2019	Spain	Cohort study	≥ 65	216 (104/112)	85	39.4%	ADL	three primary care centers	7b	
Wong [41], 2010	Canada	Baseline of Cohort study	>75	489	29	5.9%	ADL	the Montreal Unmet Needs Study	5b	
Lynch [42],2022	US	cross-sectional survey	≥ 60	4965	2569	51.7%	BADL	National Health and Nutrition Examination Surveys (NHANES)	8a	
Lynch [42], 2022	US	cross-sectional survey	≥ 60	4965	1796	36.2%	IADL	National Health and Nutrition Examination Surveys (NHANES)	8a	
Aarts [43], 2015	Iceland	Baseline of Cohort study	>65	2044	648	31.7%	ADL	Susceptibility-Reykjavik (AGES-Reykjavik) Study	7b	
Abdulazeez [44], 2021	Nigeria	Cross-sectional study	>60	201	68	33.8%	BADL	Outpatient Clinic of Aminu Kano Teaching Hospital (AKTH) Kano	9a	
Li [45], 2020	China	Baseline of Cohort study	>65	2283 (1107/1176)	372	16.3%	ADL	China Health and Retirement Longitudinal Study (CHARLS)	6b	
Storeng [46], 2020	Norway	Baseline of Cohort study	60–69	4327 (1807/2520)	55	1.3%	ADL	the Nord-Trøndelag Health Study	7b	
Koroukian [47], 2006	US	Cross-sectional study	>65	1722	638	37.0%	ADL	Ohio Cancer Incidence Surveil- lance System (OCISS)	7a	
Boeckxstaens [48], 2015	Belgium	Cohort study	≥ 80	167	34	20.4%	ADL	The BELFRAIL study	5b	
Collins [49], 2018	US	Baseline of Cohort study	>80	314 (97/217)	117	37.3%	ADL	the Hispanic Established Populations for the Epidemiologic Study of the Elderly (HEPESE)	6b	
Collins [49], 2018	US	Baseline of Cohort study	>80	314 (97/217)	202	64.3%	IADL	the Hispanic Established Populations for the Epidemiologic Study of the Elderly (HEPESE)	6b	
Grov [50], 2017	Norway	Baseline of Cohort study	≥ 70	1663	58	3.5%	ADL	The Nord-Trøndelag Health Studies (HUNT)]	6b	
Grov [50], 2017	Norway	Baseline of Cohort study	≥ 70	1663	446	26.8%	IADL	The Nord-Trøndelag Health Studies(HUNT)	6b	

a: The Agency for Healthcare Research and Quality (AHRQ)

b: The Newcastle Ottawa Scale (NOS)

### Quality assessment for methodology

A total of 32 papers was included in this study, including 17 cross-sectional [19, 21–23, 25, 28, 30–33, 35–38, 42, 44, 47] and 15 cohort studies [20, 24, 26, 27, 29, 34, 39–41, 43, 45, 46, 48–50] with a total of 71,135 individuals. Of the cross-sectional studies, 11 [19, 21–23, 31, 33, 35–38, 42, 44] (45.0%) had high-quality ratings and 6 [25, 28, 30, 32, 33, 47] (25.0%) had moderate quality ratings; of the cohort studies, 9 [24, 27, 29, 34, 39, 40, 43, 46, 47] (20.0%) and 6 [20, 26, 41, 45, 49, 50] (10.0%) of the literature rated as moderate in quality, indicating that the overall quality of the included literature was good. Nine [27, 28, 31, 36, 38–41, 44] literature explained the sampling method, seven [27, 31, 38–41, 44] used random sampling, one [28] used systematic random sampling, one [26] used multi-stage cluster random sampling, and the rest of the literature did not elaborate on the sampling method; 15 [19, 23, 26, 27, 29, 31, 35, 37, 39–43, 46, 49] articles described in detail the screening of the included population and the response rate; most of the studies did not have a follow-up, and only 8 [20, 26, 27, 29, 34, 39, 40, 46] articles had a follow-up of 1.5–10 years and described the shedding. See Table 1.

### Meta-analysis results

#### The prevalence of disability among multimorbid elderly individuals

**Fig. 2:**
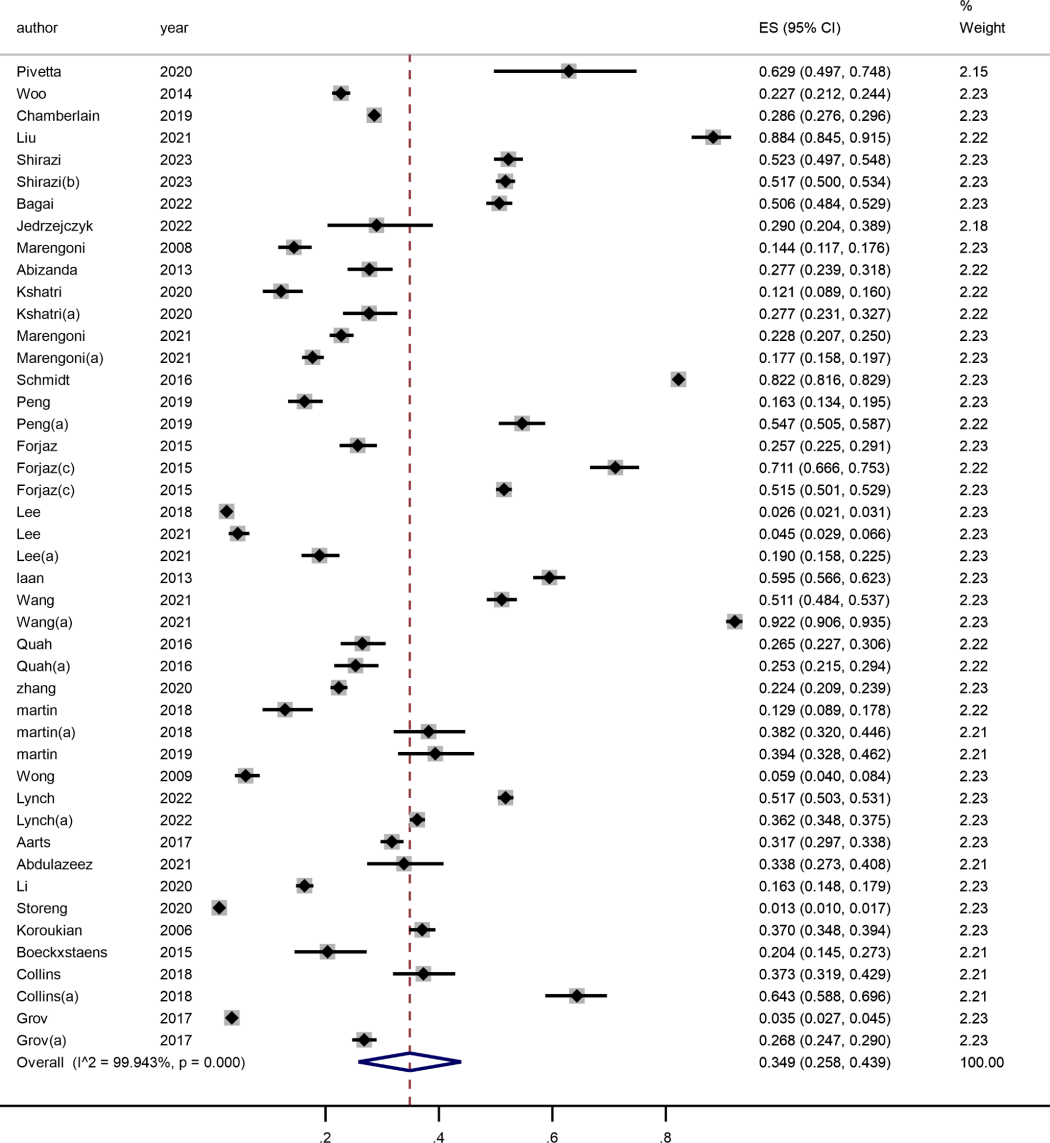
Meta-analysis of the prevalence of disability in elderly patients with multimorbidity a. Evaluate the use of different disability tools for the same population b. Evaluation of disability in different populations c. Data are from different studies

This meta-analysis comprised 32 [19–50] studies, of which 29 [19–23, 25–31, 34–50] employed ADL scales, such as BADL scales, and IADL scales, and 10 [28, 29, 31, 34, 36, 37, 39, 42, 49, 50] publications used two ADL scales. And 3 [24, 32, 33] publications utilized different measures for assessing disabilities. See Fig. 2. The studies were evaluated using a random effects model due to the studies’ significant heterogeneity (*P*<0.001, I^2^>99.9%). In older adults with multimorbidity, the prevalence of disability was 34.9% (95% *CI* = 25.8−43.9%).

### Subgroups analysis

This study has a high heterogeneity, and subgroup analysis is used to examine the origins of heterogeneity. Divided by year of publication: pre−2015, the disability rate among co-morbid patients was 27.9% [95% CI (13.7%, 42.1%)], from 2015 to 2019 the disability rate was 34.7% [95% CI (17.9%, 51.4%)], and after 2020 the disability rate was 37.2% [95% CI (23.3%, 51.0%)]. By country of investigation, the disability rate for co-morbid patients was 40.7% [95% CI (17.5%, 63.9%)] in China, 44.8% [95% CI (36.7%, 53.0%)] in the United States, 18.4% [95% CI (13.9%, 22.9%)] in Sweden, and 38.1% [95% CI (24.1%,52.0%)] in Spain, and 10.4% [95% CI (2.0%,18.8%)] in Norway. By sample size, the disability rate was 37.1% [95% CI (23.5%,51.1%)] for < 500 and 33.6% [95% CI (22.2%,45.0%)] for > 500. By gender, the rate of disability was 33.0% [95% CI (17.4%,48.6%)] for females > 50% and 22.7% [95% CI (17.9%,25.5%)] for males < 50%. By study instrument, the disability rate was 30.1% [95% CI (16.3%,44.0%)] using the ADL scale, 40.7% [95% CI (21.5%,59.9%)] using the IADL scale, 37.6% [95% CI (26.1%,49.0%)] using the BADL, and 43.9% [95% CI (26.1%,49.0%)] using the other scales. By age, the disability rate was 10.3% [95% CI (0.7%,19.49)] for 60–69 years, 21.7% [95% CI (16.2%,27.2%)] for 70–79 years, and 45.9% for ≥ 80 years. By study site, the disability rate was 21.3% [95% CI (15.8%,26.9%)] in the community and 46.7% [95% CI (24.6%,68.8%)] in hospitals. Divided by marital status, the disability rate was 16.8% [95% CI (11.5%,22.0%)] for married and 39.5% [95% CI (25.8%,53.2%)] for unmarried. When categorized by study quality, the disability rate was 39.5% [95% CI (25.8%,53.2%)] for high-quality studies and 30.8% [95% CI (17.0%,44.6%)] for moderate-quality studies. See Table 2.

**Table 2:** Subgroup analysis of disability prevalence among older patients with multimorbidity

Subgroups	Number of Studies (*n*)	Prevalence(%)	95%CI	Heterogeneity across the studies		Heterogeneity between groups (*P*-value)
				I^2^	*P*-value	
Year						
−2015	6	27.9	13.7–42.1	99.53	<0.001	*P* = 0.64
2015-	12	34.7	17.9–51.4	99.95	<0.001	
2020-	14	37.2	23.3–51.0	99.93	<0.001	
Country						
China	7	40.7	17.5–63.9	99.95	<0.001	P=<0.01
America	5	44.8	36.7–53.0	99.41	<0.001	
Sweden	2	18.4	13.9–22.9			
Spain	4	38.1	24.1–52.0	99.11	<0.001	
Norway	2	10.4	2.0−18.8			
Sample size						
<500	12	37.1	23.2–51.1	99.38	<0.001	*P* = 0.70
>500	21	33.6	22.2–45.0	99.96	<0.001	
gender						
Female > 50%	12	33.0	17.4–48.6	99.97	<0.001	*P* = 0.21
Female < 50%	3	22.7	17.9–25.5	93.33	<0.001	
Study design						
Cross-sectional study	17	43.7	29.5–57.9	99.95	<0.001	*P* = 0.01
Cohort study	15	23.7	17.5–29.9	99.65	<0.001	
Tools used						
ADL	22	30.1	16.3–44.0	99.96	<0.001	*P* = 0.75
IADL	10	40.7	21.5–59.9	99.85	<0.001	
BADL	7	37.6	26.1–49.0	98.09	<0.001	
Others	3	43.9	9.7–78.2	99.95	<0.001	
age						
60–69	4	10.3	0.7–19.9	99.95	<0.001	*P*<0.01
70–79	3	21.7	16.2–27.2			
80+	6	45.9	31.4–60.4	99.70	<0.001	
Research site						
community	6	21.3	15.8–26.9	97.41	<0.001	*P* = 0.03
Medical care institutions	7	46.7	24.6–68.8	99.83	<0.001	
Marital Status						
Married	3	16.8	11.5–22.0	97.32	<0.001	*P* = 0.07
Unmarried	3	30.7	16.4–45.1	98.97	<0.001	
Study quality						
high	16	39.5	25.8–53.2	99.93	<0.001	*P* = 0.39
moderate	16	30.8	17.0−44.6	99.95	<0.001	

### Sensitivity analysis

To ascertain if the study findings were consistent and dependable, the included literature was eliminated one at a time. The omission of a single piece of literature did not significantly alter the disability rate, according to the results of the sensitivity analysis.

### Bias analysis

**Fig. 3:**
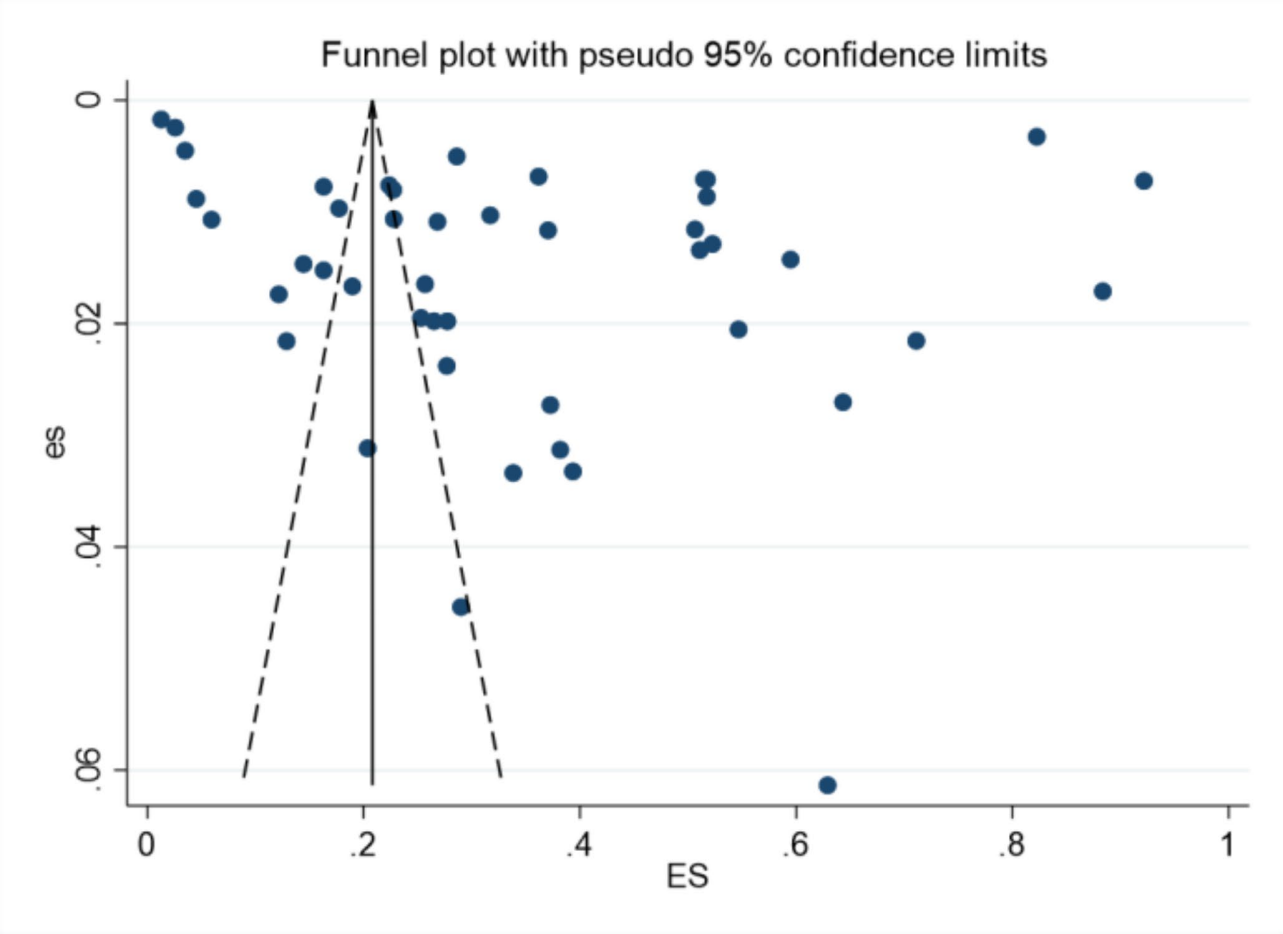
Risk bias graph

The results of the funnel plot showed that the graphical distribution was symmetrical, with a low likelihood of risk of bias. See Fig. 3. *p* = 0.03 < 0.05 in the Begg-Mazumdar’s and Egger’s tests, suggesting the possibility of publication bias.We evaluated the effect of publication bias on the results by trim and filling analysis. The results of trim and filling analysis (PR = 1.093, 95%CI = 0.988 to 1.208) did not affect the results of this study.

### Quality of evidence

We evaluated the quality of evidence for the results of the meta-analysis, which showed that the incidence of disability was of low quality.See Table 3.

**Table 3:** Evidence profile

No of studies	Design	Risk of bias	inconsistency	indrectness	imprecision	other	sample	effect	Quality
32	Observational studies	serious^a^	serious^b^	none	none	none	71,135	34.9 (95%CI = 25.8%~43.9%)	low

a: Included studies did not describe blinding, allocation concealment, etc.;

b: I^2^ > 50%, heterogeneity exists

### Conclusion

#### Findings of summary

This is the first meta-analysis to evaluate the rate of disability in elderly patients with multimorbidity. In all, 32 studies with moderate or high quality literature and dependable results were included in this analysis. The meta-analysis’s findings demonstrated that older patients with multimorbidity had a disability rate of 34.9% (95% CI = 25.8−43.9%)). The results were higher than those of middle-aged and elderly patients in Canada (27.4%) [51] and higher than the senior disability rate globally, which is 10.2% across 194 nations and territories [52]. multimorbidity may be a significant predictor of functional decline in the elderly [15, 53].

We performed subgroup analyses to determine sources of heterogeneity. The results of the subgroup analyses showed that:

1. Year of Publication: The disability rate rose with the year of publication, we suspect it has to do with the aging trend. The elderly population currently makes up 9.3% of the world’s population [54], reach 41.1% in some countries by 2050, and age is an important indication of disability [55]. On the other hand, the baseline population with disabilities rises due to better medical care, longer life expectancy, and an expanding population with multimorbidity [56]. To lessen and postpone the likelihood of disability, our response strategies to the aging trend and co-morbidity trend should be suggested earlier.
2. Demographic factors: advanced age, females, and unmarried are more likely to have disabilities. Older people are less able to successfully protect themselves against a range of hostile influences, according to prior studies [55], their physiological functions weaken, their resistance declines, and their compensating systems worsen. The functional independence of the elderly is gravely challenged by the synergistic and cumulative effects of many chronic disorders [57]. Women had a higher likelihood of being disabled, which was in line with Yau’s findings [58]. In addition to biological disparities, Zhu [59] claims that due to cultural and familial obligations, women commonly face difficulties while seeking to receive healthcare treatments. Therefore, while having a greater life expectancy than men, women may have worse health outcomes. To compensate for the differences caused by aging and gender, which are objective factors that cannot be changed, the researchers suggest that social and medical resources can be appropriately tilted, and appropriate medical assistance and financial subsidies can be provided. According to Pang’s study [60], the probability of disability is higher for people who are alone or unmarried. Based on the marital resource hypothesis, marriage has a health-protective effect that delays health deterioration, and the feeling of duty and responsibility it imparts promotes positive behaviors in old age and has an influence on health status [60]. We believe that having healthy interpersonal interactions and participating in social activities as appropriate may help to lessen the disability of celibacy people [61, 62].
3. Sample source: Compared to those in the community, hospital patients are more likely to be disabled. This is simple to understand. People with major health problems and a high risk of becoming disabled are those who frequently get primary healthcare or live in long-term care facilities. Elderly residents of the community are more physically and mentally well, have more energy for social contact, and are less prone to suffer negative outcomes. Consequently, the management of multiple medications, remote monitoring of their physical condition, diet and exercise advice, and standardized rehabilitation training are helpful assistance for people who have used healthcare services for a long time [63, 64, 76]. For community residents, we support the involvement of formal and informal caregivers in assisting patients to maintain their functional independence [65].
4. Nation: We find that the disability rate in the US, China, and Spain is around 40% higher than in Norway and Sweden. A serious issue with population aging also exists in China. Due to a mismatch in the availability and demand for healthcare services, China, the world’s largest developing country, has seen a steady increase in the burden of disabilities [66]. Spain also has one of the fastest aging populations in the world [55]. Despite being one of the best countries in the world in terms of economic strength and access to healthcare, the United States has a high rate of disability. There is a clear socioeconomic hierarchy, and those from lower socioeconomic classes have a harder time accessing better healthcare and are more likely to have health status inequalities while coping with serious sickness, according to prior research [67]. The high level of economic development, high level of education, comprehensive social security system, and a substantial proportion of public health spending may all be contributing factors to the low disability rate in places like Norway and Sweden [68].

There was high heterogeneity in this study. The researchers believe that it is related to the inconsistent standards of disability. The most commonly used is the ADL scale, while EQ−5D and Specific disability measures (24 items) have also been studied. Even articles that also use ADL have different criteria for determining disability: The first is any difficulty in basic or using tools can be defined as a disability, and the second recognizes moderate or severe dependence as a disability. In addition, the physical condition of the population, the duration of multiple diseases, and the severity of the disease can all affect the course of disability. People with two or three or more chronic diseases are more likely to become disabled than those with a single chronic disease [69]. This may be related to the deterioration of organ and tissue function exacerbated by multiple diseases. Certain patterns of comorbidities may accelerate and promote negative events. For example, psychosomatic comorbidities are more predictive of disability than other somatic comorbidities and have a higher degree of disability in the former [70–72]. Unfortunately, a similar analysis could not be performed in this study.

In recent years, the study of disability-free survival has come to be recognized as a crucial issue in the field of gerontological research [73]. With the prevalence of multimorbidity, the previous single chronic disease management model is no longer adapted to the status quo of multimorbidity. Considering the synergistic and cumulative effects of multimorbidity, countries like the United Kingdom have developed multimorbidity clinical guidelines that emphasize patient-centered healthcare services to enhance health-related quality of life [75]. A multidisciplinary integrated team treatment approach, which may offer the best diagnostic and treatment plan for patients with multimorbidity and lessen the side effects of many drugs [75, 76], has received attention from academics. Additionally, nutritional schedules, exercise recommendations, and other strategies for encouraging healthy living are thought to be effective approaches to prevent or postpone the onset of chronic illnesses and disabilities [74, 76]. Since chronic diseases require long-term care, it is envisioned that community-based staff would be incorporated as a part of the primary healthcare system to establish long-term, stable, and continuous services with the co-patient population. We urge patients to monitor their own health. Additionally, In order to effectively accompany and psychologically assist co-morbid patients, boost their adherence to rehabilitation, and decrease despair and loneliness, we expect that caregivers will be acknowledged as a crucial component of social support.

#### Strengths and limitations

The strength of this meta-analysis is that it synthesizes disability rates in older adults with multimorbidity, understands the influencing factors that exist, is the first of its kind to the best of our knowledge, and can inform the development of subsequent clinical guidelines. The study’s shortcomings are visible. First, the non-English literature was left out of this study due to language restrictions; second, some studies were not included in the meta-analysis because it was not feasible to extract data from the literature, potentially introducing bias; third, there was a sizable amount of heterogeneity in this study, but it was not brought on by the subgroups.

## Conclusion

In conclusion, around 34.9% of older co-morbid patients have varying degrees of disability. Advanced age, female gender, unmarried status, and long-term utilization of healthcare services are all likely significant factors contributing to disability. Through exercise regimens, nutritional advice, and other pharmacological management techniques, we may manage disability. The number and types of co-morbidities, and other factors could not be meta-analyzed due to the limited literature included, and it is anticipated that more high-quality, large-sample, multi-center studies will be carried out in the future to support the theory and serve as a foundation for the clinical development of an integrated, comprehensive, and personalized disease wellness program.

## Electronic supplementary material

Below is the link to the electronic supplementary material.

Supplementary Material 1

Supplementary Material 2

Supplementary Material 3

Supplementary Material 4

Supplementary Material 5

Supplementary Material 6

## Data Availability

No datasets were generated or analysed during the current study.
