# Supplementary material for: The prevalence of disability in older adults with multimorbidity: a meta-analysis
Source: Aging Clin Exp Res. 2024 Sep 10;36(1):186. doi: 10.1007/s40520-024-02835-2 (PMC11387458; doi:10.1007/s40520-024-02835-2)
Supplement: Supplementary file 1 — Supplementary Material 1 [file 40520_2024_2835_MOESM1_ESM.docx]

Highlights

1 This study synthesizes disability rates in a co-morbid population of older adults.

2 The first meta-analysis of co-morbid disability rates was performed for the elderly.

3 Disability rates in co-morbid older adults were as high as 34.9%.

4 Females, the elderly, long-term use of health care facilities,and the unmarried are more likely to have disability.
