# Supplementary material for: The prevalence of disability in older adults with multimorbidity: a meta-analysis
Source: Aging Clin Exp Res. 2024 Sep 10;36(1):186. doi: 10.1007/s40520-024-02835-2 (PMC11387458; doi:10.1007/s40520-024-02835-2)
Supplement: Supplementary file 3 — Supplementary Material 3 [file 40520_2024_2835_MOESM3_ESM.docx]

**Supplementary Table 2. Excluded literature**

| **Excluded literature** |  |
| --- | --- |
| **Full text unavailable** | 1. **Ho HE, Yeh CJ, Wei JC, Chu WM, Lee MC. Multimorbidity patterns and their relationships with incident disability and frailty among older adults in Taiwan: A 16-year, population-based cohort study. *Arch Gerontol Geriatr*. 2022;101:104688.** 2. **Lynch D H, Petersen C L, Fanous M M, et al. The relationship between multimorbidity, obesity and functional impairment in older adults[J]. Journal of the American Geriatrics Society, 2022, 70(5): 1442-1449.** 3. **Landi F, Liperoti R, Russo A, et al. Disability, more than multimorbidity, was predictive of mortality among older persons aged 80 years and older[J]. Journal of clinical epidemiology, 2010, 63(7): 752-759.** 4. **Williams J S, Egede L E. The association between multimorbidity and quality of life, health status and functional disability[J]. The American journal of the medical sciences, 2016, 352(1): 45-52.** 5. **Yuen H K, Vogtle L K. Multi-morbidity, disability and adaptation strategies among community-dwelling adults aged 75 years and older[J]. Disability and Health Journal, 2016, 9(4): 593-599.** 6. **Marventano S, Ayala A, Gonzalez N, et al. Multimorbidity and functional status in community-dwelling older adults[J]. European journal of internal medicine, 2014, 25(7): 610-616.** 7. **Casten K.S.Associations of weakness and inflammation with disability, poor cognition, and multimorbidity in older adults** 8. **Lutomski JE, Hoekstra T, Akker MVD, et al. Multimorbidity patterns in older persons and their association with self-reported quality of life and limitations in activities of daily living. *Arch Gerontol Geriatr*. 2023;115:105134. doi:10.1016/j.archger.2023.105134** |
| **Below 60 years of age** | 1. **Quiñones AR, Markwardt S, Botoseneanu A. Diabetes-Multimorbidity Combinations and Disability Among Middle-aged and Older Adults. *J Gen Intern Med*. 2019;34(6):944-951.** 2. **Pengpid S, Peltzer K, Anantanasuwong D. Bidirectional association between functional disability and multimorbidity among middle-aged and older adults in Thailand. *Front Public Health*. 2022;10:1055699. Published 2022 Dec 5.** 3. **Shi Z, Zhang Z, Shi K, et al. Association between multimorbidity trajectories and incident disability among mid to older age adults: China Health and Retirement Longitudinal Study. *BMC Geriatr*. 2022;22(1):741. Published 2022 Sep 12. doi:10.1186/s12877-022-03421-9** 4. **Aubert CE, Kabeto M, Kumar N, Wei MY. Multimorbidity and long-term disability and physical functioning decline in middle-aged and older Americans: an observational study. *BMC Geriatr*. 2022;22(1):910. Published 2022 Nov 28.** 5. **Zhou J, Wei MY, Zhang J, Liu H, Wu C. Association of multimorbidity patterns with incident disability and recovery of independence among middle-aged and older adults. *Age Ageing*. 2022;51(8):afac177.** 6. **Marmamula S, Kumbham TR, Shidhaye R, et al. Multimorbidity and multi-disability among the elderly in residential care in India: the Hyderabad Ocular Morbidity in Elderly Study (HOMES). *Sci Rep*. 2022;12(1):11779. Published 2022 Jul 11.** 7. **Zhang ZY, Yuan MQ, Shi ZX, Fang Y. *Zhonghua Liu Xing Bing Xue Za Zhi*. 2022;43(12):1893-1899.** 8. **Aguiar L B, Baccaro L F, Machado V S S, et al. Disability and multimorbidity in women older than 50 years: a population-based household survey[J]. Menopause, 2015, 22(6): 660-666.** 9. **Awuviry-Newton K, Amponsah M, Amoah D, et al. Physical activity and functional disability among older adults in Ghana: The moderating role of multi-morbidity. *PLOS Glob Public Health*. 2023;3(3):e0001014. Published 2023 Mar 8.** 10. **Wei M Y, Kabeto M U, Langa K M. Multimorbidity and Long-Term Disability and Physical Functioning Decline in Older Adults[C]//2018 Annual Research Meeting. AcademyHealth, 2018.** 11. **Zhao YW, Haregu TN, He L, et al. The effect of multimorbidity on functional limitations and depression amongst middle-aged and older population in China: a nationwide longitudinal study. *Age Ageing*. 2021;50(1):190-197.** 12. **Wang Q, Zhang S, Wang Y, Zhao D, Chen X, Zhou C. The Effect of Dual Sensory Impairment and Multimorbidity Patterns on Functional Impairment: A Longitudinal Cohort of Middle-Aged and Older Adults in China. *Front Aging Neurosci*. 2022;14:807383. Published 2022 Apr 8.** 13. **Chireh B, Essien S K, Novik N. Multimorbidity, disability, and mental health conditions in a nationally representative sample of middle-aged and older Canadians[J]. Journal of Affective Disorders Reports, 2021, 6: 100290.** |
| **Unavailability of data on disability** | 1. **St John PD, Tyas SL, Menec V, Tate R. Multimorbidity, disability, and mortality in community-dwelling older adults. *Can Fam Physician*. 2014;60(5):e272-e280.** 2. **Quiñones AR, Markwardt S, Thielke S, Rostant O, Vásquez E, Botoseneanu A. Prospective Disability in Different Combinations of Somatic and Mental Multimorbidity. *J Gerontol A Biol Sci Med Sci*. 2018;73(2):204-210. doi:10.1093/gerona/glx100** 3. **Bluethmann SM, Foo W, Winkels RM, Mama SK, Schmitz KH. Physical Activity in Older Cancer Survivors: What Role Do Multimorbidity and Perceived Disability Play?. *J Aging Phys Act*. 2020;28(2):311-319. Published 2020 Apr 24. doi:10.1123/japa.2019-0086** 4. **Yokota RT, Van der Heyden J, Nusselder WJ, et al. Impact of Chronic Conditions and Multimorbidity on the Disability Burden in the Older Population in Belgium. *J Gerontol A Biol Sci Med Sci*. 2016;71(7):903-909. doi:10.1093/gerona/glv234** 5. **Peng X, Bao X, Xie Y, et al. The mediating effect of pain on the association between multimorbidity and disability and impaired physical performance among community-dwelling older adults in southern China. *Aging Clin Exp Res*. 2020;32(7):1327-1334. doi:10.1007/s40520-019-01324-1** 6. **Lu FP, Chang WC, Wu SC. Geriatric conditions, rather than multimorbidity, as predictors of disability and mortality among octogenarians: A population-based cohort study. *Geriatr Gerontol Int*. 2016;16(3):345-351. doi:10.1111/ggi.12480** 7. **Bernardes GM, Mambrini JVM, Lima-Costa MF, Peixoto SV. Perfil de multimorbidade associado à incapacidade entre idosos residentes na Região Metropolitana de Belo Horizonte, Brasil [Multimorbidity profile associated with disability among the elderly living in the Metropolitan Region of Belo Horizonte, Brazil]. *Cien Saude Colet*. 2019;24(5):1853-1864. Published 2019 May 30. doi:10.1590/1413-81232018245.17192017** 8. **Salive ME. Multimorbidity in older adults. *Epidemiol Rev*. 2013;35:75-83. doi:10.1093/epirev/mxs009** 9. **Garin N, Olaya B, Moneta MV, et al. Impact of multimorbidity on disability and quality of life in the Spanish older population. *PLoS One*. 2014;9(11):e111498. Published 2014 Nov 6. doi:10.1371/journal.pone.0111498** 10. **Bleijenberg N, Zuithoff NPA, Smith AK, de Wit NJ, Schuurmans MJ. Disability in the Individual ADL, IADL, and Mobility among Older Adults: A Prospective Cohort Study. *J Nutr Health Aging*. 2017;21(8):897-903. doi:10.1007/s12603-017-0891-6** 11. **da Rosa PPS, Marques LP, Corrêa VP, De Oliveira C, Schneider IJC. Is the combination of depression symptoms and multimorbidity associated with the increase of the prevalence of functional disabilities in Brazilian older adults? A cross-sectional study. *Front Aging*. 2023;4:1188552. Published 2023 May 23. doi:10.3389/fragi.2023.1188552** 12. **Sheridan PE, Mair CA, Quiñones AR. Associations between prevalent multimorbidity combinations and prospective disability and self-rated health among older adults in Europe. *BMC Geriatr*. 2019;19(1):198. Published 2019 Jul 27. doi:10.1186/s12877-019-1214-z** 13. **Su P, Ding H, Zhang W, et al. The association of multimorbidity and disability in a community-based sample of elderly aged 80 or older in Shanghai, China. *BMC Geriatr*. 2016;16(1):178. Published 2016 Oct 27. doi:10.1186/s12877-016-0352-9** 14. **Eckerblad J, Theander K, Ekdahl A, et al. To adjust and endure: a qualitative study of symptom burden in older people with multimorbidity[J]. Applied Nursing Research, 2015, 28(4): 322-327.** 15. **Juul-Larsen H G, Andersen O, Bandholm T, et al. Differences in function and recovery profiles between patterns of multimorbidity among older medical patients the first year after an acute admission—An exploratory latent class analysis[J]. Archives of Gerontology and Geriatrics, 2020, 86: 103956.**   **16.Cheung JTK, Yu R, Wu Z, Wong SYS, Woo J. Geriatric syndromes, multimorbidity, and disability overlap and increase healthcare use among older Chinese. *BMC Geriatr*. 2018;18(1):147. Published 2018 Jun 25. doi:10.1186/s12877-018-0840-1.**  **17.Qiao Y, Liu S, Li G, et al. Longitudinal Follow-Up Studies on the Bidirectional Association between ADL/IADL Disability and Multimorbidity: Results from Two National Sample Cohorts of Middle-Aged and Elderly Adults. *Gerontology*. 2021;67(5):563-571. doi:10.1159/000513930**  **18.Botoseneanu A, Markwardt S, Quiñones AR. Multimorbidity and Functional Disability among Older Adults: The Role of Inflammation and Glycemic Status - An Observational Longitudinal Study. *Gerontology*. 2023;69(7):826-838. doi:10.1159/000528648**  **19.Jackson CA, Jones M, Tooth L, Mishra GD, Byles J, Dobson A. Multimorbidity patterns are differentially associated with functional ability and decline in a longitudinal cohort of older women. *Age Ageing*. 2015;44(5):810-816. doi:10.1093/ageing/afv095**  **20.Wallace E, McDowell R, Bennett K, Fahey T, Smith SM. Comparison of count-based multimorbidity measures in predicting emergency admission and functional decline in older community-dwelling adults: a prospective cohort study. *BMJ Open*. 2016;6(9):e013089. Published 2016 Sep 20. doi:10.1136/bmjopen-2016-013089**  **21.Fisher K, Griffith LE, Gruneir A, Kanters D, Markle-Reid M, Ploeg J. Functional limitations in people with multimorbidity and the association with mental health conditions: Baseline data from the Canadian Longitudinal Study on Aging (CLSA). *PLoS One*. 2021;16(8):e0255907. Published 2021 Aug 11. doi:10.1371/journal.pone.0255907**  **22.Sieber S, Roquet A, Lampraki C, Jopp DS. Multimorbidity and Quality of Life: The Mediating Role of ADL, IADL, Loneliness, and Depressive Symptoms. *Innov Aging*. 2023;7(4):igad047. Published 2023 Jun 4. doi:10.1093/geroni/igad047**  **23.Ansari S, Anand A, Hossain B. Multimorbidity and depression among older adults in India: Mediating role of functional and behavioural health. *PLoS One*. 2022;17(6):e0269646. Published 2022 Jun 7. doi:10.1371/journal.pone.0269646**  **24.Landré B, Gil-Salcedo A, Jacob L, et al. The role of age, sex, and multimorbidity in 7-year change in prevalence of limitations in adults 60-94 years. *Sci Rep*. 2022;12(1):18270. Published 2022 Oct 31. doi:10.1038/s41598-022-23053-8**  **25.Ćwirlej-Sozańska A, Wiśniowska-Szurlej A, Wilmowska-Pietruszyńska A, Sozański B. Determinants of ADL and IADL disability in older adults in southeastern Poland. *BMC Geriatr*. 2019;19(1):297. Published 2019 Oct 31. doi:10.1186/s12877-019-1319-4**  **26.Kumar M, Kumari N, Chanda S, Dwivedi LK. Multimorbidity combinations and their association with functional disabilities among Indian older adults: evidence from Longitudinal Ageing Study in India (LASI). *BMJ Open*. 2023;13(2):e062554. Published 2023 Feb 6. doi:10.1136/bmjopen-2022-062554**  **27.Hou C, Ping Z, Yang K, et al. Trends of Activities of Daily Living Disability Situation and Association with Chronic Conditions among Elderly Aged 80 Years and Over in China. *J Nutr Health Aging*. 2018;22(3):439-445.**  **28.Makovski TT, Le Coroller G, Putrik P, et al. Role of clinical, functional and social factors in the association between multimorbidity and quality of life: Findings from the Survey of Health, Ageing and Retirement in Europe (SHARE). *PLoS One*. 2020;15(10):e0240024. Published 2020 Oct 20.**  **29.Raina P, Gilsing A, Mayhew AJ, Sohel N, van den Heuvel E, Griffith LE. Individual and population level impact of chronic conditions on functional disability in older adults. *PLoS One*. 2020;15(2):e0229160. Published 2020 Feb 20. doi:10.1371/journal.pone.0229160**  **30.Botes R, Vermeulen KM, Correia J, Buskens E, Janssen F. Relative contribution of various chronic diseases and multi-morbidity to potential disability among Dutch elderly. *BMC Health Serv Res*. 2018;18(1):24. Published 2018 Jan 15. doi:10.1186/s12913-017-2820-0**  **31.Leme DEDC, Thomaz RP, Borim FSA, Brenelli SL, Oliveira DV, Fattori A. Survival of elderly outpatients: effects of frailty, multimorbidity and disability. Estudo do impacto da fragilidade, multimorbidade e incapacidade funcional na sobrevida de idosos ambulatoriais. *Cien Saude Colet*. 2019;24(1):137-146.**  **32.Waterhouse P, van der Wielen N, Banda PC, Channon AA. The impact of multi-morbidity on disability among older adults in South Africa: do hypertension and socio-demographic characteristics matter?. *Int J Equity Health*. 2017;16(1):62. Published 2017 Apr 8.** |
| **Not relevant to the topic** | **1. Hermans H, Evenhuis H M. Multimorbidity in older adults with intellectual disabilities[J]. Research in Developmental Disabilities, 2014, 35(4): 776-783.**  **2.Hussain R, Wark S, Janicki MP, Parmenter T, Knox M. Multimorbidity in older people with intellectual disability. *J Appl Res Intellect Disabil*. 2020;33(6):1234-1244.**  **3.Vetrano DL, Damiano C, Tazzeo C, et al. Multimorbidity Patterns and 5-Year Mortality in Institutionalized Older Adults. *J Am Med Dir Assoc*. 2022;23(8):1389-1395.e4.**  **4.Drewes YM, den Elzen WP, Mooijaart SP, de Craen AJ, Assendelft WJ, Gussekloo J. The effect of cognitive impairment on the predictive value of multimorbidity for the increase in disability in the oldest old: the Leiden 85-plus Study. *Age Ageing*. 2011;40(3):352-357. doi:10.1093/ageing/afr010**  **5.Malik MA. Functional disability among older adults in India; a gender perspective. *PLoS One*. 2022;17(9):e0273659. Published 2022 Sep 14.**  **6.McCarron M, Swinburne J, Burke E, McGlinchey E, Carroll R, McCallion P. Patterns of multimorbidity in an older population of persons with an intellectual disability: results from the intellectual disability supplement to the Irish longitudinal study on aging (IDS-TILDA). *Res Dev Disabil*. 2013;34(1):521-527.**  **7.Espeland M A, Justice J N, Bahnson J, et al. Eight-year changes in multimorbidity and frailty in adults with type 2 diabetes mellitus: associations with cognitive and physical function and mortality[J]. The Journals of Gerontology: Series A, 2022, 77(8): 1691-1698.**  **8.Axmon A, Björkman M, Ahlström G. Hospital readmissions among older people with intellectual disability in comparison with the general population. *J Intellect Disabil Res*. 2019;63(6):593-602.**  **9.Schoufour JD, Oppewal A, van der Maarl HJK, et al. Multimorbidity and Polypharmacy Are Independently Associated With Mortality in Older People With Intellectual Disabilities: A 5-Year Follow-Up From the HA-ID Study. *Am J Intellect Dev Disabil*. 2018;123(1):72-82.**  **10.Watkins LV, Henley W, Sun JJ, et al. Tackling increased risks in older adults with intellectual disability and epilepsy: Data from a national multicentre cohort study. *Seizure*. 2022;101:15-21.**  **11.Hu WH, Liu YY, Yang CH, et al. Developing and validating a Chinese multimorbidity-weighted index for middle-aged and older community-dwelling individuals. *Age Ageing*. 2022;51(2):afab274.**  **12.Calderón-Larrañaga A, Vetrano DL, Welmer AK, Grande G, Fratiglioni L, Dekhtyar S. Psychological correlates of multimorbidity and disability accumulation in older adults. *Age Ageing*. 2019;48(6):789-796.**  **13.Hilderink H B M, Plasmans M H D, Snijders B E P, et al. Accounting for multimorbidity can affect the estimation of the Burden of Disease: a comparison of approaches[J]. Archives of Public Health, 2016, 74: 1-16.**  **14.Cesari M, Pérez-Zepeda M U, Marzetti E. Frailty and multimorbidity: different ways of thinking about geriatrics[J]. Journal of the American Medical Directors Association, 2017, 18(4): 361-364.**  **15.Kandola A, Stubbs B, Koyanagi A. Physical multimorbidity and sedentary behavior in older adults: Findings from the Irish longitudinal study on ageing (TILDA)[J]. Maturitas, 2020, 134: 1-7.**  **16.Singer L, Green M, Rowe F, et al. Trends in multimorbidity, complex multimorbidity and multiple functional limitations in the ageing population of England, 2002–2015[J]. Journal of comorbidity, 2019, 9: 2235042X19872030.**  **17.Calderón-Larrañaga A, Vetrano D L, Welmer A K, et al. Psychological correlates of multimorbidity and disability accumulation in older adults[J]. Age and ageing, 2019, 48(6): 789-796.**  **18.Jacob L, Shin JI, Kostev K, et al. Prospective Association between Multimorbidity and Falls and Its Mediators: Findings from the Irish Longitudinal Study on Ageing. J Clin Med. 2022;11(15):4470. Published 2022 Jul 31.**  **19.Amankwaa I, Nelson K, Rook H, Hales C. Association between body mass index, multi-morbidity and activities of daily living among New Zealand nursing home older adults: a retrospective analysis of nationwide InterRAI data. BMC Geriatr. 2022;22(1):62. Published 2022 Jan 19.**  **20.Calderón-Larrañaga A, Vetrano DL, Ferrucci L, et al. Multimorbidity and functional impairment-bidirectional interplay, synergistic effects and common pathways. J Intern Med. 2019;285(3):255-271.** |
