# Supplementary material for: The prevalence of disability in older adults with multimorbidity: a meta-analysis
Source: Aging Clin Exp Res. 2024 Sep 10;36(1):186. doi: 10.1007/s40520-024-02835-2 (PMC11387458; doi:10.1007/s40520-024-02835-2)
Supplement: Supplementary file 4 — Supplementary Material 4 [file 40520_2024_2835_MOESM4_ESM.docx]

**Supplementary Table 1. Search strategies.**

| **Database** | **Search** | **#** |
| --- | --- | --- |
| **Pubmed** | (("activities of daily living"[Title/Abstract] OR "disability"[Title/Abstract] OR "disabled"[Title/Abstract] OR "ADL"[Title/Abstract]) AND (("older adults"[Title/Abstract] OR "older population"[Title/Abstract] OR "aged"[Title/Abstract] OR "elderly"[Title/Abstract] OR "geriatric"[Title/Abstract]))) AND ("multimorbidity"[Title/Abstract] OR "multiple chronic conditions"[Title/Abstract]) | **616** |
| **Web of Science** | ( “activities of daily living” OR “disability” OR “disabled” OR “ADL” )(Abstract) AND ( “multimorbidity” OR “multiple chronic conditions” )(Abstract) AND ( “older adults” OR “older population” OR “aged” OR “elderly” OR “geriatric” ) (Abstract) | **718** |
| **Embase** | ('multimorbidity':ti,ab,kw OR 'multiple chronic condition':ti,ab,kw) AND ('disability':ti,ab,kw OR 'dialy life activity':ti,ab,kw OR 'adl':ti,ab,kw OR 'disabled':ti,ab,kw) AND ('older people':ti,ab,kw OR 'elderly':ti,ab,kw OR 'senior citizen':ti,ab,kw OR 'older adults':ti,ab,kw OR 'aged':ti,ab,kw OR 'geriatric':ti,ab,kw) | **599** |
| **CINAL** | AB ( “activities of daily living” OR “disability” OR “disabled” OR “ADL” ) AND AB ( “multimorbidity” OR “multiple chronic conditions” ) AND AB ( “older adults” OR “older population” OR “aged” OR “elderly” OR “geriatric” ) | **768** |
| **Cochrane** | ((“activities of daily living” OR “disability” OR “disabled” OR “ADL”)):ti,ab,kw AND ((“multimorbidity” OR “multiple chronic conditions”)):ti,ab,kw AND ((“older adults” OR “older population” OR “aged” OR “elderly” OR “geriatric”)): ti, ab, Kw. | **122** |
