# Supplementary material for: The prevalence of disability in older adults with multimorbidity: a meta-analysis
Source: Aging Clin Exp Res. 2024 Sep 10;36(1):186. doi: 10.1007/s40520-024-02835-2 (PMC11387458; doi:10.1007/s40520-024-02835-2)
Supplement: Supplementary file 6 — Supplementary Material 6 [file 40520_2024_2835_MOESM6_ESM.docx]

**Supplementary Fig 2. Trim and filling analysis Chart**
